# An anatomical study of porcine peripheral nerve and its potential use in nerve tissue engineering

**DOI:** 10.1111/joa.12341

**Published:** 2015-07-21

**Authors:** Leyla Zilic, Philippa E Garner, Tong Yu, Sabiniano Roman, John W Haycock, Stacy-Paul Wilshaw

**Affiliations:** 1 Department of Materials Science and Engineering, University of SheffieldSheffield, UK; 2 School of Biomedical Sciences, Faculty of Biological Sciences, University of LeedsLeeds, UK

**Keywords:** extracellular matrix, peripheral nerve, porcine, ultrastructure

## Abstract

Current nerve tissue engineering applications are adopting xenogeneic nerve tissue as potential nerve grafts to help aid nerve regeneration. However, there is little literature that describes the exact location, anatomy and physiology of these nerves to highlight their potential as a donor graft. The aim of this study was to identify and characterise the structural and extracellular matrix (ECM) components of porcine peripheral nerves in the hind leg. Methods included the dissection of porcine nerves, localisation, characterisation and quantification of the ECM components and identification of nerve cells. Results showed a noticeable variance between porcine and rat nerve (a commonly studied species) in terms of fascicle number. The study also revealed that when porcine peripheral nerves branch, a decrease in fascicle number and size was evident. Porcine ECM and nerve fascicles were found to be predominately comprised of collagen together with glycosaminoglycans, laminin and fibronectin. Immunolabelling for nerve growth factor receptor p75 also revealed the localisation of Schwann cells around and inside the fascicles. In conclusion, it is shown that porcine peripheral nerves possess a microstructure similar to that found in rat, and is not dissimilar to human. This finding could extend to the suggestion that due to the similarities in anatomy to human nerve, porcine nerves may have utility as a nerve graft providing guidance and support to regenerating axons.

## Introduction

The peripheral nervous system (PNS) is comprised of nerves, enclosed bundles of long fibres or axons and neurons, which connect the central nervous system to the rest of the body (Saladin, 2011). The primary function of the PNS is to allow for movement, sensation and changes in behaviour to be undertaken in response to external or internal stimuli. Peripheral nerves in the lower limb are composed of sensory, motor and sympathetic fibres. The sciatic nerve, situated in the posterior compartment of the leg, is the largest nerve beginning in the lower back and runs down towards the lower limb. Its function consists of providing motor innervation to the muscles of the posterior aspect of the thigh and those of the leg and foot, and sensory innervation to the skin of the lateral aspect of the leg and almost all of the foot (Gray et al. 1995). The sciatic nerve divides into two terminal branches – the tibial and common peroneal nerve, with the tibial nerve being the larger of the branches. The tibial nerve provides motor innervation to the muscles of the posterior compartment of the leg and sensory innervation to the posterior aspect of the leg, via its contribution to the sural nerve and the sole of the foot. The peroneal nerve provides motor innervation to the muscles of the lateral compartment of the leg, which innervates the foot, and sensory innervation to the distal section and dorsum of the foot (McCrory et al. 2002). The sural nerve, formed by the junction of the medial sural cutaneous nerve with the peroneal branch, is a small superficial sensory nerve providing innervation to the posterior calf, lateral ankle, heel and foot (Riedl & Frey, 2013).

When considering injuries to peripheral nerves, it is reported that several hundred thousand such injuries occur each year in Europe (300 000 cases annually; Mohanna et al. 2003). Peripheral nerve injuries are more common than spinal cord injuries, and over 50 000 surgical procedures are performed annually in the USA to repair damaged peripheral nerves (Noble et al. 1998). Current treatment is comprised of either direct end-to-end surgical suturing of the damaged nerve ends or the use of an autologous nerve graft. Suturing is limited to the repair of small defects or gaps in the nerve. For longer nerve gaps, the current ‘gold standard’ treatment is autologous grafting – using a sensory nerve such as the sural nerve to replace the injured tissue. There are limitations to using this method, most notably donor site morbidity, chronic pain, and a lack of suitable donor nerve tissue. Due to a relatively small diameter, multiple sural nerve segments may be placed side by side to match the width of the nerve being replaced (Dahlin, 2008). In addition, sural nerves also possess smaller fascicular patterns (i.e. the number and size of fascicles), which may not match the fascicular patterns of the nerve being grafted (Meek & Coert, 2002). Considering these limitations, there are clinical requirements for better approaches to aid nerve regeneration (Schmidt & Leach, 2003).

One such approach is the use of nerve guide conduits (NGCs), which entubulate and reconnect the proximal and distal nerve stumps. NGCs function by providing support and a physical substrate designed to mimic the nerve extracellular matrix (ECM) and therefore facilitate axon regrowth. Commercially available NGCs are either composed of naturally derived materials such as collagen, or synthetic biodegradable polymers such as poly (glycolic acid). NGCs have the potential to reduce the need for autologous nerve; however, they are only suitable for short gap injuries and the maximum regeneration distance is typically limited to 10–15 mm irrespective of NGC material or design (Bell & Haycock, 2012). A plausible explanation for this is the absence of physical guidance for regenerating axons at the relevant length scales (∼50 μm) or the presence of factors including a Schwann cell basal lamina and ECM (Spivey et al. 2012). It is believed that the 3D ultrastructure, surface topology and composition of the ECM are essential in providing precise guidance for axonal regeneration. Evidence suggests that residual cellular components can negate the tissue remodelling capacity of the scaffold by providing cues that influence cell migration, proliferation and differentiation, as well as inducing a constructive host tissue remodelling response (Crapo et al. 2011). NGCs are not able to fully replicate this highly complex matrix and therefore their ability to facilitate axon regrowth is limited.

Tissue and organ decellularisation has been proposed as a method to create scaffolds for regenerative medicine applications. The process of decellularisation aims to remove all of the native cells from a given tissue without adversely affecting its biochemical and mechanical properties. A resulting decellularised graft should retain a native ECM, which does not elicit an immune response, and may provide a native microenvironment containing cell-adhesive and growth-supporting properties. It is hypothesised that such a graft will support axon regrowth for the repair of small gap nerve defects (Whitlock et al. 2009).

Human donor nerves have been used in nerve repair. Avance® Nerve Graft, a commercial available decellularised human nerve allograft, is reported to better support nerve regeneration compared with commercially available NGCs (Whitlock et al. 2009). However, the supply of human nerve tissue for decellularisation and use as a graft material is extremely limited in the UK, and therefore research has focused on the use of xenogeneic tissue due to ease of harvesting and availability. For small gap repair, xenogeneic nerve tissue from rat, rabbit and pigs have been evaluated (Gutmann & Sanders, 1943; Osawa et al. 1990; Hudson et al. 2004; Whitlock et al. 2009; Zhang et al. 2010). Rat sciatic nerve has been extensively characterised and evaluated for use as a nerve graft due to its ease of harvest and wide availability (Table 1; Osawa et al. 1990; Hudson et al. 2004; Whitlock et al. 2009; Wang et al. 2014). However, rat sciatic nerves have limitations on the size of nerve that can be obtained. In contrast, it is hypothesised that porcine nerves are anatomically more similar to human nerves. Other pig tissues and organs closely approximate their human counterparts; cross-linked porcine heart valves for example have been widely used clinically. Moreover, porcine nerves have been considered as suitable for trauma studies of the facial nerve (Barrs et al. 1991). In addition, porcine nerve tissue may have the potential to be used for longer and more specific nerve gap injuries due to their size, length, motor and sensory similarities compared with human nerves (Moore et al. 2011).

**Table 1:** A summary of xenogeneic nerves that have been used for decellularisation studies

Nerve type	Origin	References
Sciatic nerve	Sprague–Dawley rats	Hudson et al. (2004)
Sciatic nerve	Rat	Whitlock et al. (2009)
Sciatic nerve	Rat	Osawa et al. (1990)
Sciatic nerve	Lewis rats	Jesuraj et al. (2011)
Sciatic nerve	Rat	Wang et al. (2014)
Sciatic nerve	Sprague–Dawley rats	Kim et al. (2004)
Intercostal nerves	Porcine	Zhang et al. (2010)
Sural nerve	*Macaca fascicularis* primate	Hess et al. (2007)
Nerve segments (tibial and peroneal)	Rhesus monkey	Hu et al. (2007)

To the best of the authors' knowledge there have been very few studies evaluating the potential of porcine peripheral nerves as grafts to repair short and long gap defects. However, in order to use porcine nerves clinically, an understanding of the anatomy and physiology of porcine peripheral nerves is required, and this is currently absent from the literature. Therefore, the aim of the present work was to study the anatomical organisation, structure and characteristics of the major peripheral nerves in the porcine hind leg.

## Materials and methods

### Dissection of peripheral nerves in the lower limb

Male adult Wistar rats were killed by a Schedule 1 method (following regulations of the Animals Scientific Procedures Act 1986, UK). The dissection of rat sciatic peripheral nerves was carried out using the method described by Kaewkhaw et al. (2012). Yorkshire pigs (24–26 weeks old) were obtained from a local abattoir (J. Penny, Leeds, UK) within 24 h of slaughter. The peripheral nerves were dissected from the porcine leg. The sciatic, tibial, common peroneal and sural nerves were isolated and dissected, with initial reference to the anatomy of human nervous system anatomy of the lower leg. The sciatic nerve was dissected from the posterior compartment of the leg. The sciatic nerve divided into two terminal branches; the tibial and common peroneal nerve. The tibial nerve was observed to travel in the posterior section of the leg and the peroneal nerve in the lateral section. The sural nerve was dissected by a longitudinal incision made from the popliteal fossa along the posterior midline and towards the posterior-inferior aspect of the lateral malleolus. Excess fat and connective tissue was removed from the nerve samples, and tissues washed three times in phosphate-buffered saline solution (PBS; Oxoid, Basingstoke, UK) containing 0.1% (w/v) ethylene diamine tetra acetic acid (EDTA; VWR) to remove excess blood and tissue fluid.

### Characterisation of peripheral nerves in the lower limb

Native porcine and rat nerve tissue was cut into 1 cm segments, dissected from either end of the nerve (*n* = 3) and fixed in 10% (v/v) neutral buffered formalin (NBF) for 24 h. Samples were then dehydrated in an automated tissue processer (Leica TP1020) before being embedded into paraffin wax (VWR) to form histology blocks. Transverse sections (6 μm) of each nerve sample were cut using a rotary microtome (Leica RM 2125 RTF). Sections were de-waxed and dehydrated before staining by submerging sequentially in xylene (2 × 10 min), 100% ethanol (3 × 2 min), 70% (v/v) ethanol (2 min) and then water (3 min). Sections were viewed using an Olympus BX51 microscope and images captured using an Olympus XC50 digital camera (with olympus soft imaging solutions software). The number and size of the nerve fascicles present within each peripheral nerve was analysed using imagej (NIH, USA).

### Haematoxylin and eosin staining of peripheral nerves

Haematoxylin and eosin (H&E; Raymond A Lamb Ltd, UK) staining of rat sciatic and porcine peripheral nerves (sciatic, sciatic branches, sural and cutaneous branches) was undertaken to evaluate how the histoarchitecture and size of the nerve fascicular pattern differed between the species in regards to branching of the lower limb. Samples were immersed in Harris haematoxylin (Thermo Fisher Scientific, UK; 1 min) and rinsed under tap water for blueing (3 min). Slides were then immersed into eosin Y (VWR International; 3 min), dehydrated, cleared and mounted using DPX mountant before being viewed under Kohler illumination.

### Characterisation of porcine peripheral nerve ECM

Porcine sciatic branches (tibial and common peroneal) and sural nerve were stained using Picro sirius red and Millers elastic stain (Raymond A Lamb) for collagen and elastin, respectively. The same slide was used to stain for both the collagen and elastin. Alcian Blue Periodic Acid Schiff's stain (ABPAS; Raymond A Lamb) was used to localise sulphated glycosaminoglycans (GAGs). Porcine sciatic branches (tibial and common peroneal) were fluorescently immunolabelled using antibodies against laminin and fibronectin.

### Staining porcine peripheral nerves for collagen and elastin

Samples were immersed in 5% (w/v) potassium permanganate (Thermo Fisher Scientific) for 5 min and then rinsed with distilled water, and then submerged into 1% (w/v) oxalic acid for 2 min, rinsed with water for a further 4 min and submerged in 95% (v/v) ethanol and 70% (v/v) ethanol for 1 min, respectively. Samples were then stained for 1 h with Millers' stain (Raymond A Lamb) and rinsed with 95% (v/v) ethanol, 70% (v/v) ethanol and distilled water, respectively. Samples were subsequently stained with Weigert's iron haematoxylin (Atom Scientific, UK) for 10 min and rinsed with distilled water for 30 s for blueing. Samples were then stained with 0.1% (w/v) Picro-Sirius Red (Sigma Aldrich, UK) for 1 h, rinsed with distilled water and blot dried. Sections were dehydrated, cleared and mounted using DPX mountant before being viewed under Kohler and polarised illumination.

### Staining porcine peripheral nerves for sulphated GAGs

Samples were immersed into 1% (w/v) ABPAS (pH 2.5; Atom Scientific) for 1 min and rinsed with distilled water. The slides were then immersed in 0.1% (w/v) periodic acid solution (Sigma) for 5 min and rinsed three times with distilled water. The slides were then immersed in Schiff's reagent (Sigma) for 15 min and rinsed with distilled water for 5 min; cell nuclei were stained with haematoxylin (Gills Number 3 haematoxylin; Sigma) for 90 s. Samples were blued using tap water, dehydrated, cleared and mounted using DPX mountant before being viewed using an upright microscope under Kohler illumination.

### Immunolabelling of porcine peripheral nerves for laminin and fibronectin

Tissue sections were circled with a hydrophobic marker and gently permeabilised with 0.1% (v/v) Triton X-100 diluted in PBS for 20 min. Samples were then incubated with 7.5% (w/v) bovine serum albumin (BSA; Sigma) diluted in PBS at room temperature for 60 min, followed by washing once with 1% (w/v) BSA in PBS. Laminin: nerve tissue samples were incubated with primary rabbit anti-laminin antibody (polyclonal IgG; 0.01 mg mL^−1^; Sigma, LN393, in 1%; (v/v) BSA) at 4 °C overnight, followed by washing three times with PBS for 5 min and then incubated with secondary FITC-conjugated anti-rabbit IgG [Abcam UK; ab97050, 1: 100 (v/v)] at room temperature in the dark for 1 h. Each section was washed three times with PBS for 5 min and counterstained with 300 nm 4, 6-diamidino-2-phenylindole dihydrochloride (DAPI) in PBS and incubated for 20 min in the dark at room temperature. Sections were finally washed three times with PBS for 5 min and then immersed in PBS. Images were captured using long focal distance (3.5 mm) ‘water dipping’ objective lenses (10 ×/0.3 and 20 ×/0.5; Zeiss Achroplan) and a Zeiss LSM510 META upright/inverted confocal microscope [xenon arc lamp to excite FITC (*λ*_ex_ = 495 nm/*λ*_em_ = 515 nm)]. Nuclei were visualised using *λ*_ex_ = 510 nm/*λ*_em_ = 610–650 nm). Fibronectin: nerve samples were incubated with a primary anti-fibronectin rabbit polyclonal antibody (Sigma; F3648) in 1% (w/v) BSA at 1: 400 (v/v) dilution) at 4 °C overnight. Each sample was washed three times with PBS for 5 min each and incubated with secondary FITC-conjugated anti-rabbit IgG [1: 100 (v/v) dilution] at room temperature in the dark for 1 h. Sections were subsequently washed three times with PBS for 5 min each and counterstained with 300 nm DAPI for 20 min in the dark at room temperature. Samples were then washed three times with PBS for 5 min and then immersed in PBS. Images were captured using long focal distance (3.5 mm) ‘water dipping’ objective lenses (10 ×/0.3 and 20 ×/0.5; Zeiss Achroplan) and a Zeiss LSM510 META confocal microscope [xenon arc lamp to excite FITC (*λ*_ex_ = 495 nm/*λ*_em_ = 515 nm)]. Nuclei were visualised using (*λ*_ex_ = 510 nm/*λ*_em_ = 620–650 nm).

### Detection of hydroxyproline and sulphated sugars

Samples of the tibial and common peroneal porcine sciatic branches were lyophilised to a constant weight prior to biochemical assay for collagen and sulphated sugars content.

#### Hydroxyproline assay

The procedure adopted was based on the method described by Woessner and subsequently modified by Edwards & O'Brien (1980). The hydroxyproline concentration of acid-hydrolysed samples (6 m HCl at 120 °C for 4 h) was determined by interpolation from a trans-4-hydroxy-l-proline standard curve. The total collagen content was calculated by using a hydroxyproline to collagen ratio of 1: 7.69 (Ignat'eva et al. 2007).

#### Sulphated sugar assay

The sulphated sugar content of papain-digested samples (6 m HCl at 120 °C for 4 h) was determined using dimethylene blue (Sigma) according to Farndale et al. (1986). The GAG content was determined by interpolating values from a standard curve of chondroitin sulphate B (Sigma).

### Assessment of mechanical properties of porcine peripheral nerves

Peroneal and tibial nerve samples were cut to 1 cm in length and each of their diameters was individually measured. Hydrated samples were clamped on a tensiometer (BOSE Electroforce Test Instruments, Minnesota, USA) using a 450 N load cell, and a ramp test was run at a rate of 0.1 mm s^−1^. The first failure point (or plateau) was used to calculate the ultimate tensile strength, and the initial linear gradient was taken as the Young's modulus. For all specimens the mean ultimate stress, strain at failure and Young's modulus were determined from the initial length and area of the specimens.

### Nuclei labelling of porcine peripheral nerve

Porcine peripheral nerves (sciatic branches and sural nerve) were labelled with DAPI, which identifies cell nuclei (blue) and enables visual localisation of all cells within the tissue. Samples were incubated with 300 nm DAPI for 10 min in the dark, washed with PBS × 3 for 10 min in the dark, and mounted with glass coverslips using DABCO: glycerol mountant (Sigma) and stored in the dark. Nuclei were imaged using an upright fluorescent microscope and a DAPI filter (*λ*_ex_ = 510 nm/*λ*_em_ = 620–650 nm). Images were captured using a digital camera (Image pro Plus v 5.1).

### Labelling porcine peripheral nerve tissue with nerve growth factor receptor p75 (NGFR P75)

To determine the location of putative Schwann cells, porcine sciatic branches were immunolabelled for NGFR P75. Initially, antigen retrieval of samples was carried out by adding Proteinase K (Dako) for 30 min at room temperature. Endogenous peroxidase in the tissue was blocked by incubating samples in 3% (v/v) hydrogen peroxide (Sigma) diluted in PBS for 10 min at room temperature. Tissue sections were washed using tap water for 3 min and then with TRIS-buffered saline (TBS; pH 7.4) solution for 10 min. Dual endogenous enzyme block (25 μL; Ultra Vision kit; Thermo-Scientific) was added to each sample and incubated for 10 min. Primary anti-human CD271 NGFR P75 antibody (mouse monoclonal IgG; BioLegend, 345102; 0.2 μg mL^−1^) was added to each section and incubated in a humidified atmosphere at room temperature for 60 min. Samples were then washed × 2 with TBS containing 0.05% (w/v) Tween 20 (TBS-T) and finally × 2 with TBS for 10 min. Visualisation of antibody labelling was achieved by addition of 15 μL chromagen to each section and incubating for 10 min at room temperature. The substrate chromagen was made up by adding 20 μL of liquid diaminobenzide (DAB) Plus Chromagen (Ultra Vision Kit; Thermo-Scientific) to 1 mL DAB Plus Substrate (Ultra Vision Kit; Thermo-Scientific). Samples were then washed 4 × using distilled water and counterstained using Harris' haematoxylin for 10 s. The samples were then rinsed under tap water for 1 min for blueing. Samples were dehydrated before being mounted using DPX Mountant and viewed under Kohler illumination (Olympus BX 51 microscope). Isotype control antibodies (IgG1; Dako) were used to verify antibody specificity.

### Labelling porcine peripheral nerve tissue with S100β

To determine the presence of Schwann cells more specifically, porcine sciatic branches were immunolabelled for the glial marker S100β. Initially, antigen retrieval of samples was carried out by adding 100 μL of 0.17% trypsin working solution [1% (v/w) calcium chloride (VWR) solution added to 0.5% (v/v) porcine trypsin solution (Sigma)] to each section and incubated at 37 °C for 20 min and left to cool for a further 10 min at room temperature. Samples were then incubated with 7.5% (w/v) BSA diluted in PBS at room temperature for 60 min, followed by washing once with 1% (w/v) BSA in PBS. The nerve tissue samples were incubated with primary rabbit anti-S100β antibody [polyclonal IgG; Abcam, ab868, 1: 50 (v/v) in 1%; (v/v) BSA] at 4 °C overnight, followed by washing three times with PBS for 5 min and then incubated with secondary FITC-conjugated anti-rabbit IgG [Abcam UK, ab97050, 1: 500 (v/v)] at room temperature in the dark for 1 h. Each section was washed three times with PBS for 5 min and counterstained with 300 nm DAPI in PBS and incubated for 20 min in the dark at room temperature. Sections were finally washed three times with PBS for 5 min and imaged using a Zeiss LSM510 META upright/inverted confocal microscope [xenon arc lamp to excite FITC (*λ*_ex_ = 495 nm/*λ*_em_ = 515 nm)]. Nuclei were visualised using excitation at *λ*_ex_ = 510 nm and emission capture at *λ*_em_ = 610–650 nm.

## Results and discussion

Peripheral nerve injuries occur in both the upper and lower extremities due to motor vehicle crashes, industrial accidents, as well as gunshot and stab wounds (Noble et al. 1998). Peripheral nerve injury in the upper extremity is most commonly reported, with the most affected nerve including ulnar, digital and radial. In the lower limb the sciatic and peroneal nerves are the most frequently affected nerves (Eser et al. 2009). In this study the identification and characterisation of porcine peripheral nerves located in the lower limb is reported. The rat sciatic nerve was also included in the study as a control.

**Fig. 1:**
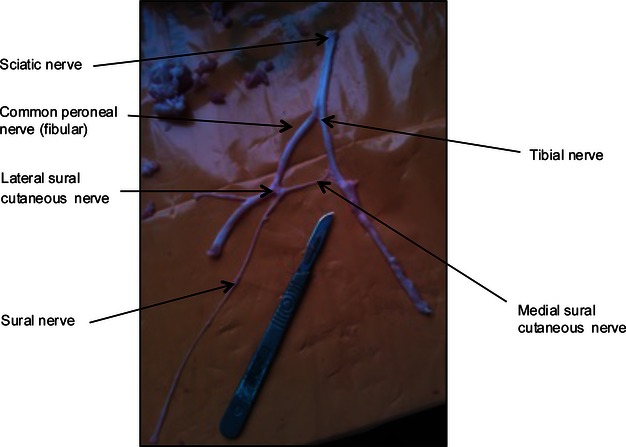
Peripheral nerves dissected from the posterior section of the porcine hind limb. The sciatic nerve divides into the tibial and common peroneal nerve. Two cutaneous branches, the medial and lateral nerves, branch off from the tibial and peroneal nerve, respectively, to form the sural nerve.

Porcine peripheral nerves were dissected from the posterior section of the porcine hind limb. The sciatic divided into two branches of the tibial and common peroneal nerve (Fig. 1). Both the tibial and peroneal nerves run caudal to the stifle joint with the tibial running into the muscle. Two cutaneous branches, the medial and lateral, branch off from the tibial and peroneal nerve, respectively, to form the sural nerve. The general nerve branching observed was consistent with similar findings in the peripheral nerve anatomy of humans as well as other mammals such as rat (Sunderland & Ray, 1948; Schmalbruch, 1986). In addition, the length and dimensions of the porcine nerves were found to be comparable to that of human nerves (Gustafson et al. 2012).

**Fig. 2:**
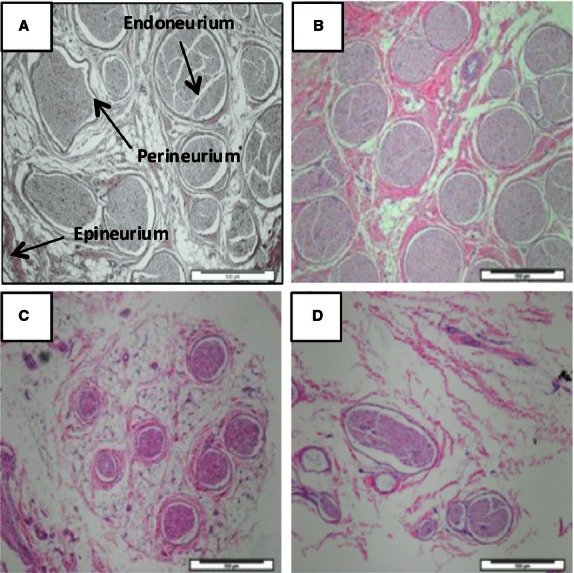
Histoarchitecture of transverse porcine peripheral nerve sections stained with haemotoxylin and eosin. The sciatic nerve (A) and its branches (peroneal and tibial nerves) (B) have numerous nerve fascicles packed closely together within the epineurium. The sural nerve (C) and the cutaneous branches (D) have much smaller and sparsely distributed nerve fascicles within the epineurium. Scale bar: 500 μm.

**Fig. 3:**
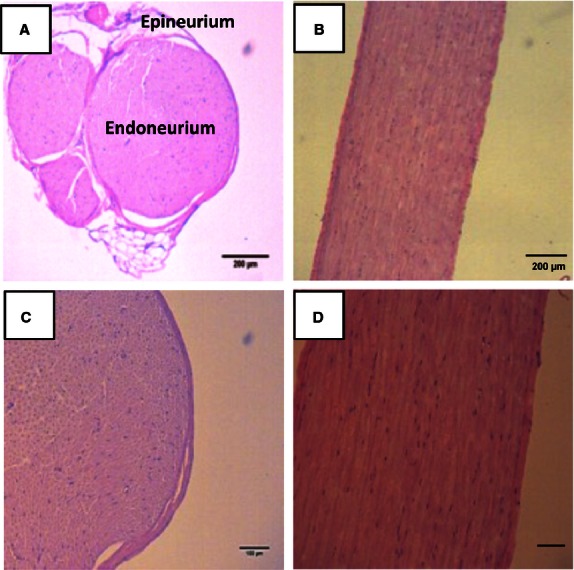
Histoarchitecture of (A,C) transverse rat peripheral nerve and (B,D) longitudinal orientation, stained using haemotoxylin and eosin. Transverse sections show the rat sciatic nerve fascicles containing endoneurium and perineurium. Longitudinal sections show nucleated cells (blue) aligning amongst the collagen fibrils. Scale bar: 200 μm (A,B); 100 μm (C,D).

The histoarchitecture of porcine and rat peripheral nerve sections showed variation in the funiculi pattern within each nerve segment as they branch (Figs 2 and 3). The sciatic nerve and its branches (peroneal and tibial nerves) have numerous nerve fascicles packed closely together within the epineurium, whilst the sural nerve and the cutaneous branches have much smaller and sparsely distributed nerve fascicles. These data were quantified and showed the porcine sciatic nerve to have an average of 11 fasicles with an average area of 150 ± 34.18 mm^2^, whilst its branches have on average 20 fasicles with an average area of 94.08 ± 12.28 mm^2^. The sural cutaneous branch had an average of six fasicles with an average area of 68.97 ± 35.69 mm^2^, and the sural nerve had an average seven fasicles with an average area of 47.93 ± 6.510 mm^2^. In contrast, the rat sciatic nerve had an average of three fasicles with an average area of 157.3 mm^2^ (Table 2).

**Table 2:** The average number and area of fascicles present in porcine and rat peripheral nerves

Nerve	Average number of fascicles	Area of fascicles (mm^2^)
Porcine sciatic	11	150.45 ± 34.18
Porcine sciatic branches	20	94.08 ± 12.28
Porcine sural cutaneous branch	6	68.97 ± 35.69
Porcine sural	7	47.93 ± 6.51
Rat sciatic	3	157.30 ± 80.16

The sciatic nerve had an average 11 fasicles with an average area of 150 ± 34.18 mm^2^ (*n* = 10); sciatic branches (peroneal/tibial nerve) had on average 20 fasicles with an average area of 94.08 ± 12.28 mm^2^ (*n* = 27); the sural cutaneous branch had an average of six fasicles with an average area of 68.97 ± 35.69 mm^2^; the sural branch had an average of seven fasicles with an average area of 47.93 ± 6.51 mm^2^. The rat sciatic nerve had an average of three fasicles with an average area of 157.30 mm^2^ (*n* = 3). Three porcine legs were used to obtain the figures in this experiment.

The fasicular pattern observed in the porcine nerves was similar to that reported for human studies (Sunderland, 1990; Chentanez et al. 2006; Ugrenovic et al. 2013; Ugrenović, 2014). When mapping the branches from the sciatic nerve, the fascicular patterns within the individual nerves have previously been shown to differ in respect to the arrangement, size and number. Sunderland & Ray (1948) reported no constant or characteristic pattern in regards to the number and size of the funiculi, which varied greatly from nerve-to-nerve and individual-to-individual at any given level (Sunderland & Ray, 1948). Interestingly, a study by Ugrenović (2014) showed a significant difference in the average number of fascicles between the tibial and common peroneal nerve. The common peroneal nerve had a significantly lower number of fascicles; however, there was no significant difference in the average value of fascicular area or diameter between the two nerves (Ugrenović, 2014).

The internal nerve structure has substantial relevance in terms of the clinical outcome of nerve lesions, as well as the surgical repair of nerve injuries. In direct nerve repair the surgeon aligns the fascicles by matching similar looking ones in the stumps to prevent misalignment in addition to using epineurial blood vessels (Stewart, 2003). Similarly in nerve grafting, attention to the fascicular pattern is thought to be of importance in achieving good functional recovery (Stewart, 2003). In turn, an appropriately matched nerve graft would lead to a more accurate technical repair, minimal sutures and potentially less scarring.

A study conducted by Burks et al. (2014) highlighted the challenge of insufficient donor nerve graft with a specific focus on human sciatic nerve transection requiring autologous sural nerve graft. The authors reported that a considerable degree of variability existed in the diameter of the fascicles and cross-sectional area of the sural nerve harvested, in comparison with the sciatic nerve. The study also reiterated the fact that small sensory nerves such as the sural nerve do not provide sufficient material for grafting and that allogeneic intercostal nerves (common peroneal or tibial) are preferred (Burks et al. 2014). This approach was reported by Mackinnon et al. (2001) where the tibial nerve was reconstructed with allograft tissue.

Mechanical testing of the porcine branches in Table 3 showed the peroneal nerve to have a Young's modulus of 7.75 ± 1.26 MPa, ultimate tensile stress of 1.23 ± 0.13 MPa and strain at failure of 0.23 ± 0.08. The tibial nerve had a Young's modulus of 7.43 ± 1.69 MPa, ultimate tensile stress of 0.87 ± 0.29 MPa and strain at failure of 0.164 ± 0.05. The peroneal nerve had a significantly higher stress value in comparison to the tibial nerve; however, both nerve types were comparable in terms of modulus and stress. In comparison with other studies, the ultimate tensile stress and strain at failure values of the porcine nerves were found to be slightly lower than that of rat sciatic nerve, reported as 2.7 MPa and 81%, respectively (Rydevik et al. 1990; Borschel et al. 2003). A reason for the high strain reported in rat sciatic nerve is that rat nerve has a lower total ECM per unit volume, resulting in a more pliable material (Borschel et al. 2003). The lower ultimate tensile stress found in the porcine nerves in comparison with rat may be attributed to the sciatic nerve branches being tested rather than the sciatic nerve itself.

**Table 3:** Mechanical testing of porcine peroneal and tibial nerves

Nerve	Young's modulus (MPa)	Ultimate tensile stress (MPa)	Strain at failure
Peroneal	7.75 ± 1.26	1.23 ± 0.13	0.23 ± 0.08
Tibial	7.43 ± 1.69	0.87 ± 0.29	0.16 ± 0.05 *

The peroneal nerve had a Young's modulus of 7.75 ± 1.26 MPa, ultimate tensile stress of 1.23 ± 0.13 MPa and strain at failure of 0.23 ± 0.08. The tibial nerve had a Young's modulus of 7.43 ± 1.69 MPa, ultimate tensile stress of 0.87 ± 0.29 MPa and strain at failure of 0.164 ± 0.05. Four porcine legs were used to obtain the figures for this experiment.

All data are mean ± standard deviation. Peroneal nerve was *n* = 8 and tibial nerve *n* = 11 (*P < 0.05).

The sciatic nerves in both porcine and rat possess larger fascicles in comparison to its branches (Figs 2 and 3; Table 2). It was initially demonstrated by Sunderland & Bradley (1961) that there are more fascicles and larger cross-sectional area of extrafasicular connective tissue present in regions when the nerve passed a joint, such as the sciatic nerve. This led to the later suggestion that this was a protective feature by which vulnerable areas of nerves resisted mechanical injury (Sunderland & Bradley, 1961). A study by Phillips et al. (2004) also concluded that sciatic nerves exhibit more strain in the joint region; however, it was further concluded that this was a result of the complex tissue architecture rather than fascicle number (Mason & Phillips, 2011). In addition, it has been reported that nerve stiffness is greater in long nerve sections and in sections with numerous branch points, such as the sciatic nerve (Millesi et al. 1995). Future studies may be informative by considering the mechanical properties of the porcine sciatic nerve. Other factors that contribute to the overall mechanical properties of peripheral nerves include the internal fluid pressure maintained by the impermeable perineurium (Low et al. 1977) and ECM components such as collagen and elastin (Ushiki & Ide, 1990; Tassler et al. 1994).

In terms of clinical applications, the mechanical properties of peripheral nerves are very important. Nerve graft coaptations are tension free by design; however, *in situ* stress is always present in peripheral nerves and varies with joint position as previously mentioned (Sunderland & Bradley, 1961). Properties such as suture holding ability and the ability to maintain a mechanically robust interface with the native nerve stump are critical for axon regeneration (Borschel et al. 2003).

**Fig. 4:**
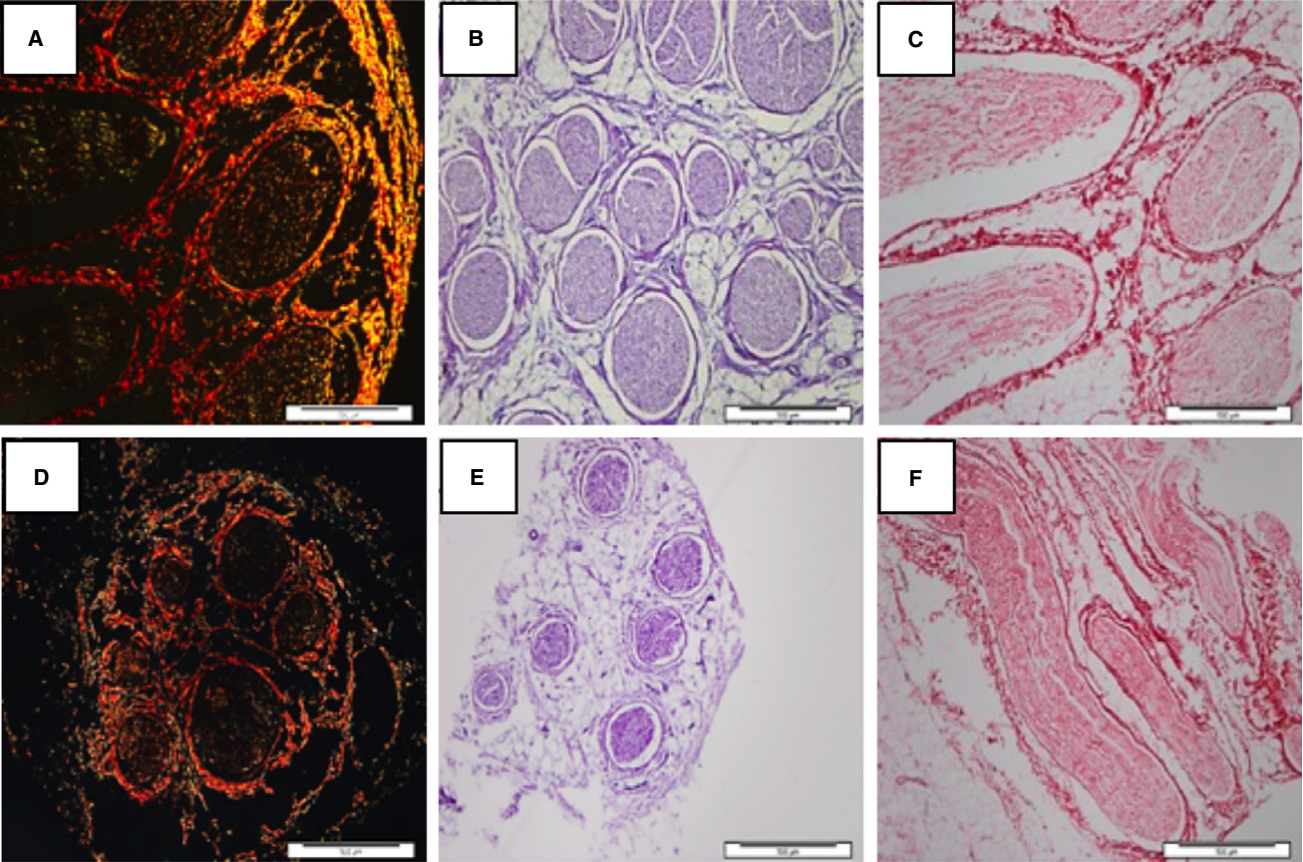
Histological staining of ECM components in porcine peripheral nerves. Sciatic nerve branches (A–C) and the sural nerve (D–F) were stained with Picro sirius red for identification of collagen (A,D), ABPAS for GAGs (B,E) and Millers stain for elastic fibres (C,F). Staining revealed that collagen was located within the endoneurium, perineurium and epineurium (red identifies thicker collagen fibres and green, thinner newly formed fibres). GAGs were found within the endoneurium (purple), although no presence of elastic fibres (blue/black) was detected in either nerve type. Scale bar: 500 μm.

The ECM of the porcine nerves was characterised herein (Fig. 4). The porcine nerves were stained for elastic fibres using Millers stain (Fig. 4C,F); however, fibres (blue/black) were not detected. Reports have stated that distinguishing the presence of elastin from collagen is difficult, as both collagen and elastin co-label when using traditional histochemical techniques such as Weigert and Verhoeff-VanGiesson (Tassler et al. 1994). Tassler et al. (1994) did, however, combat this problem by using immunospecific stains, and found elastin to be present in all three connective layers of the peripheral nerve, predominately around the perineurium and to a lesser extent in the epineurium and within the endoneurium.

A study by Sunderland (1968) suggested that elastin fibres were responsible for the nerve exhibiting a visco-elastic behaviour in their ability to respond to a range of motion of the joints it crosses and its resilience in withstanding the traction demands imposed by injury (Sunderland, 1968). However, a study carried out Tassler et al. (1994) reports that the percentage of elastic fibres found in nerve was relatively small in comparison to collagen, and has therefore suggested that the elastic properties of peripheral nerves are more likely to be due to elastin in the first phase of the curve (strain < 20%) and collagen thereafter.

Staining of the porcine nerves (sciatic branches and sural) with Picro sirius red revealed collagen to be a major component of the ECM (Fig. 4A,D), with a large number of collagen fibres located around the epineurium (red), perineurium (red) and within the endoneurium (green). It has been postulated that observed differences in the polarised colour correlates with collagen fibre thickness, and in turn enables collagen isotype to be deduced (Junqueira et al. 1982). Thus, collagen type I fibres as well as newly laid down collagen are revealed as thick, strongly birefringent yellow/red fibres in the epineurium, whilst collagen type III is present in the form of thin, weakly birefringent green fibres in the endoneurium (Junqueira et al. 1982).

A similar collagen distribution has been observed in human, rat and rabbit studies (Junqueira et al. 1979). A study by Kaemmer et al. (2010) compared the collagen distribution between the epineurium and endoneurium between human, porcine and rat nerves, and found that collagen distribution in human tissue was 14.6/3.5 (epineurium/endoneurium), rat 28.4/3.3, and in porcine tissue 19.4/2.9. Although there is a slight variation between the species, a similar pattern was reported by Seyer et al. (1977) on human femoral nerve, describing 81% of collagen type I being located in the epineurium and 19% collagen type III in the endoneurium.

From the current results, in Table 4 the total collagen content in the porcine peripheral nerves was found to contain 1212 ± 194.8 μg mg^−1^ collagen. In comparison, the human sural nerve was found to contain 2500 μg mg^−1^ and the rat sciatic nerve 123 μg mg^−1^ (Myers et al. 1977). The high amount of collagen found in the nerves is plausible, as Seyer et al. (1977) concluded that collagen accounts for approximately 49% of all proteins in human peripheral nerve tissue. In contrast to collagen, levels of sulphated GAGs in Table 4 found in the porcine nerves were relatively lower, with a value of 17 ± 1.3 μg mg^−1^. Characterisation of the sciatic branches and sural nerve using ABPAS revealed GAGs (which stained purple) predominately found within the endoneurium (Fig. 4B,E). Only a small literature exists regarding the characterisation of GAGs in peripheral nerve; however, a study by Junqueira et al. (1981) reported that the human sciatic nerve contains 0.32 μg mg^−1^. While a detailed comparison of porcine nerve GAG is therefore difficult, the difference with human nerve is of note. Interestingly, a study by Chandrasekaran & Bachhawat (1969) characterised GAGs in the peripheral nerve of monkeys and found hyaluronic acid to be the major GAG present, comprising 63% of the peripheral nerve, while chondroitin-4-sulphate comprised 16%. The remaining components were 7.5% heparin sulphate, 5.2% chondroitin-6-sulphate and 8% hyaluronidase resistant galactosamine-GAG.

**Table 4:** Quantification of sulphated GAGs and collagen content in porcine sciatic branches

Assay	Native nerve (μg mg^−1^ dry weight)
Collagen (hydroxyproline × 7.69)	1206 ± 196
Sulfated proteoglycans	16.15 ± 1.3

The nerves were found to contain a total of 17 ± 1.3 μg mg^−1^ GAGs (*n* = 9) and 1212 ± 196 μg mg^−1^ collagen (*n* = 8).

The importance of hyaluronic acid in nerve regeneration is relatively unknown; however, it has been suggested that it assists with fibrin organisation, which may facilitate a pathway for cellular and axonal ingrowth during the acellular fibrin matrix phase of peripheral nerve regeneration (Wang et al. 1998). Other studies suggest that chondroitin sulphated proteoglycans inhibit the neurite promoting activity of laminin, and this has a negative impact on nerve regeneration (Zuo et al. 1998). Therefore, in recognition of the different types of GAGs residing within the peripheral nerve and their roles in nerve regeneration, it would be interesting to investigate the non-sulphated GAGs to obtain a true representation of the amount of GAGs present.

**Fig. 5:**
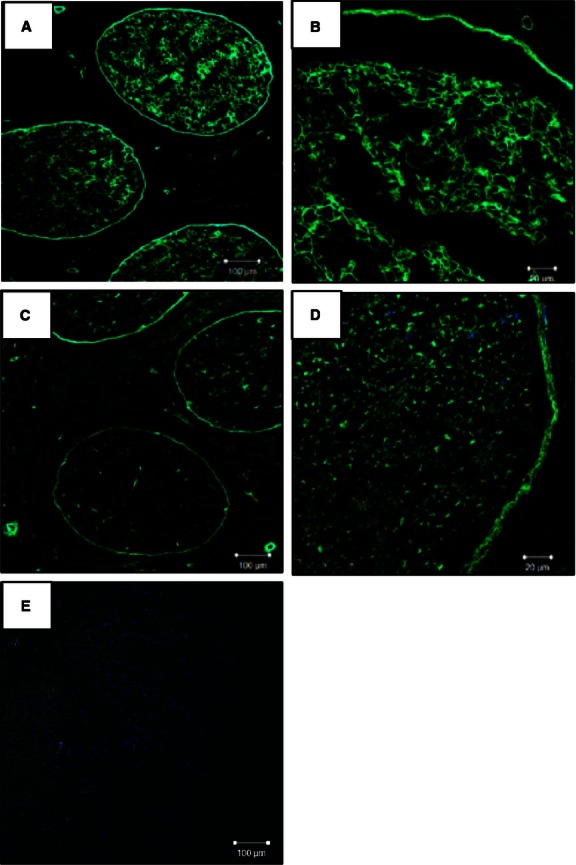
Immunolabelling for laminin and fibronectin within porcine sciatic branches. Laminin was found predominantly around the perineurium and within the endoneurium (A,B). Fibronectin was found predominantly around the perineurium, and some labelling identified with the epineurium and within the endoneurium (C,D). Control with only secondary phalloidin-FITC-conjugated anti-rabbit IgG (for fibronectin and laminin, respectively; E). Scale bar: 100 μm and 20 μm.

**Fig. 6:**
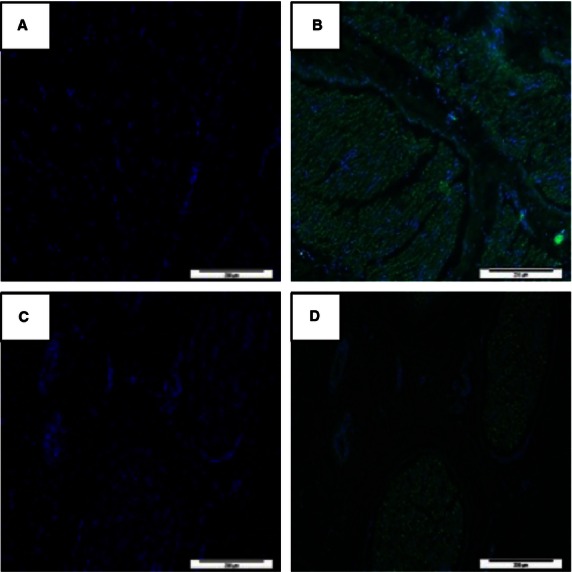
Nuclei labelling (DAPI) revealed cells within the endoneurium and perineurium of both the sciatic branches (A,B) and sural nerve (C,D). A sparse distribution of cells was also found in the epineurium and surrounding arterioles. Cell nuclei are shown in blue and surrounding ECM in green. Scale bar: 500 μm.

**Fig. 7:**
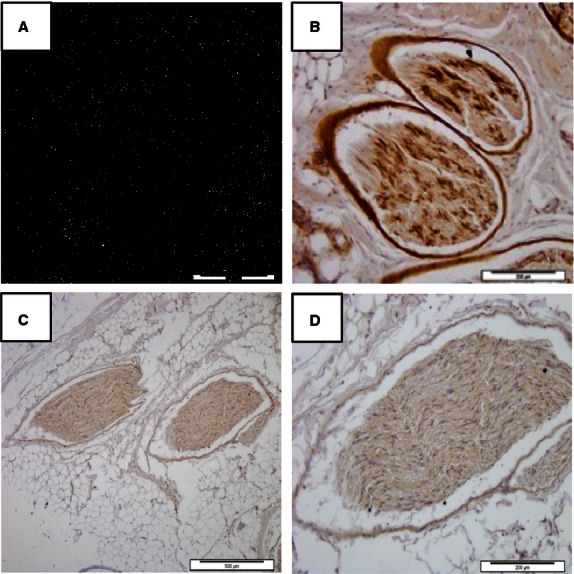
Porcine peripheral nerve tissue immunolabelled for NGFR P75 to identify and localise Schwann cells/p75-positive fibroblasts. Cells were found to be located within the endoneurium and around the perineurium of the nerve fascicle (A,B). Negative controls (C,D). Scale bar: 500 μm and 200 μm.

**Fig. 8:**
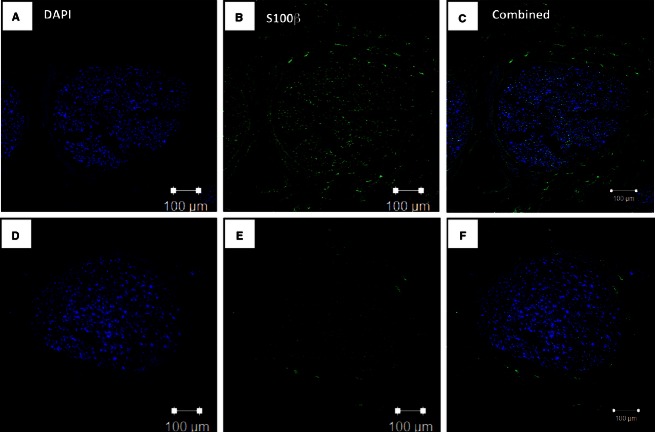
Porcine peripheral nerve tissue immunolabelled for S100β to identify and localise Schwann cells. Samples are co-labelled with DAPI (nuclei). Cells were found to be located within the endoneurium of the nerve fascicle (A–C). Negative controls with DAPI and secondary antibody only (D–F) Scale bar: 100 μm.

In this study, laminin and fibronectin were also identified (Fig. 5), as well as peripheral nerve cells (Figs 6 and 7). Laminin and fibronectin were selected as important constituents of the peripheral nerve ECM as well as their role in nerve regeneration (Gao et al. 2013). Laminin, which is located within the endoneurium and around the perineurium (Fig. 5A,B), makes up the basal lamina along with collagen and plays an essential role in enhancing axonal growth (Gao et al. 2013). Fibronectin, located around the perineurium and within the epineurium (Fig. 5C,D), has been shown to promote Schwann cell growth and motility, thereby enhancing regeneration of injured nerves (Ahmed et al. 2003). Labelling of total nuclei (DAPI; Fig. 6) confirmed cells to be located within the endoneurium and around the perineurium, with a sparse distribution of cells in the epineurium and surrounding arterioles of both the sciatic branches (Fig. 6A,B) and sural nerve (Fig. 6C,D). Positive immunolabelling of NGFR P75 confirmed that Schwann cells were located primarily around the perineurium and within the endoneurium (Fig. 7). However, the perineurial cells may be NGFR-positive fibroblast cells, therefore the tissue was also immunolabelled for the glial marker S100β, localising the Schwann cells solely to the endoneurium (Fig. 8). This is a plausible explanation as one of the functions of Schwann cells is the myelination of axons, which are located within the endoneurium (Bhatheja & Field, 2006).

In summary, the present study reports on a detailed description on the anatomy of porcine peripheral nerves in the lower limb. The results demonstrate that porcine nerves are more comparable to human nerves than rat in terms of anatomical, biochemical and cellular components. The study highlights anatomical differences of the nerve as it branches, as well as the mechanical properties of the sciatic nerves. The porcine nerve fascicles were characterised for the presence of collagen, GAGs, laminin and fibronectin together with Schwann cells, all of which play an essential role in terms of structural support as well as nerve regeneration. The authors suggest that the similarities between porcine and human nerve may allow for the clinical use of porcine nerves as grafts following nerve injury.
